# Remedy for Earache

**Published:** 1885-11

**Authors:** 


### Remedy for Earache.

We can recommend, from our personal experience, says the *Therapeutic Gazette*, an effectual means of administering chloroform in this complaint, and one which is absolutely devoid of danger. This is too loosely fill the bowl of a common clay pipe with cotton batting, upon which pour as much chloroform as it will retain without dripping. This done, insert the end of the stem carefully into the ear, and placing the opening of the bowl in the mouth blow gently the vapor of chloroform against the tympanum. We have found this to be an exceedingly effectual relief for earache of children, uncomplicated, of course, with inflammatory disturbances.—*Medical Times*.

